# Supplementary material for: Are carbon emissions trading and green financial instruments synergistic? -Comprehensive quantitative research based on content analysis
Source: PLoS One. 2024 Mar 7;19(3):e0298601. doi: 10.1371/journal.pone.0298601 (PMC10919601; doi:10.1371/journal.pone.0298601)
Supplement: S1 Data — (ZIP) [file pone.0298601.s001.zip › Data/地级市Z_F工作报告.zip!/║╙▒▒/╠╞╔╜╩╨/└┤╫╘╣1⁄2╓┌║┼í╛┬φ┐╦╩2╛▌═°í┐_2016.docx]

各位代表：

现在，我代表唐山市人民政府向大会作工作报告，请连同《唐山市国民经济和社会发展第十三个五年规划纲要(草案)》一并审议，并请市政协委员和列席会议的同志提出意见。

一、“十二五”时期和2015年工作回顾

过去五年，是唐山发展史上极不寻常的五年。面对复杂多变的宏观环境和艰巨繁重的发展任务，全市上下在省委、省政府和市委坚强领导下，坚持稳中求进、改革创新，顽强拼搏、奋力攻坚，顺利完成“十二五”规划主要目标任务，开创了唐山经济社会发展和各项事业新局面。

综合经济实力跃上新台阶。预计全市地区生产总值由2010年的4469亿元增加到2015年的6100亿元，扣除价格因素，年均增长8.1%，人均地区生产总值由5.9万元增加到7.8万元、年均增长7.3%，总量继续保持全省首位。一般公共预算收入由195.8亿元增加到335亿元、年均增长11.4%，规模以上工业增加值达到2710亿元、年均增长9.4%，固定资产投资4560亿元、年均增长14.3%，社会消费品零售总额2130亿元、年均增长13.5%。

结构调整迈出新步伐。三次产业结构由9.4:58.2:32.4调整优化为9.4:55.2:35.4。钢铁、焦化、水泥等传统产业增加值占规模以上工业比重由51.5%下降到37%，装备制造业占规模以上工业比重由9.7%提高到20%。服务业增加值是2010年的1.5倍，高新技术产业增加值年均增长16.7%。市级以上农业龙头企业达到384家，农业产业化经营率提高到67.5%。

沿海开发取得新进展。沿海地区生产总值、一般公共预算收入占全市比重分别达到30.5%和38.1%。曹妃甸区正式设立，国家级经济技术开发区、石化产业基地成功获批，中日韩循环经济示范基地获准建设，全省首家综合保税区封关运营。京冀(曹妃甸)协同发展示范区建设全面启动。华润曹妃甸电厂、中储粮油脂基地等一批重大项目竣工投产或开工建设。

改革开放取得新成效。行政审批、国有企业、财税金融、医药卫生、农业农村等重点领域改革不断深化，市县政府机构改革全面完成，市本级行政审批事项精简率为63.6%。15家国有企业改制退出，民营经济增加值年均增长9.2%。组建金融发展集团，燕赵财险等金融机构落户唐山。双向开放打开新局面，省级以上开发区(园区)达到36家，实际利用外资和对外投资分别累计完成65.1亿美元、12.4亿美元，是“十一五”的1.8倍和3.8倍。

城乡面貌呈现新变化。全市常住人口城镇化率达到57.4%，比2010年提高6.6个百分点。城市综合承载能力和管理水平进一步提高，新建、改造城市道路151条，集中供热面积比2010年增加4226万平方米，城市自来水和燃气普及率达到100%、污水集中处理率达到95%，居全国同等城市领先水平。新农村建设扎实推进，完成1079个省级重点村改造提升任务。

综合交通体系实现新跨越。唐山港新增开放泊位32个，与70多个国家和地区160个港口通航，货物综合吞吐能力达到5.12亿吨。津秦客专、唐山客车线建成投运，铁路、公路、高速公路运营通车里程分别达到1266公里、17841公里和610公里，在全省率先实现“县县通高速”。三女河机场旅客吞吐量大幅跃升。

生态建设得到新加强。坚定不移压能、降耗、减排、增绿，累计压减炼铁产能1087万吨、炼钢产能2357万吨，削减煤炭消费量1079万吨，单位GDP能耗下降率为26.1%，2015年PM2.5平均浓度较2013年下降26.1%。推进山水林田湖生态修复工程，累计造林绿化101万亩，森林覆盖率达到35.6%。

人民生活水平实现新提高。全市居民人均可支配收入23330元，居全省第1位。其中，城镇居民人均可支配收入31200元，居全省第2位;农村居民人均可支配收入13960元，居全省第1位。城镇累计新增就业49万人，城乡低保标准分别比2010年提高61.3%和130.5%，新农合参合率达到98.5%。教育、文化、体育、医疗卫生等社会事业蓬勃发展，精神文明和民主法治建设全面推进，荣膺河北省首个“全国文明城市”。安全生产和食品药品监管持续加强，信访维稳工作扎实有效，社会大局和谐稳定。民族宗教、外事侨务、防震减灾、老区建设、人口计生、妇女儿童、史志档案、气象、国土、旅游、审计、统计、质监、人防、法制、邮政、老龄、残疾人、国防动员和双拥共建等工作取得了新成绩。

各位代表!刚刚过去的2015年，是全市上下攻坚突破、砥砺奋进的一年。“十二五”中后期，受外部冲击和自身结构的叠加影响，唐山发展经历了一个从未有过的艰难时期，经济持续下行，指标增长乏力，长期积累的结构性矛盾全面凸显。在极其严峻的形势下，我们按照“32239”总体思路，顶住压力，保持定力，抓当前、谋长远，既“砍柴”、又“磨刀”，力促经济在困境中开拓、在转型中发展。经过去年的艰苦努力，全市经济顶住了下行的巨大压力，出现了企稳向好的发展势头，转型升级迈出坚实步伐。

一是着力打好经济下行阻击战。制定出台稳增长遏制工业经济下滑、政银企对接等系列政策措施，加强对困难企业和成长性企业的帮扶服务，力促经济平稳健康发展。预计全年地区生产总值增长5%，固定资产投资增长10%，规模以上工业增加值增长4%，一般公共预算收入增长3.5%，社会消费品零售总额增长9%， 进出口总值完成139.1亿美元，实际利用外资14.2亿美元，全市经济实现了新常态下的新发展。

二是着力以新增长点推动经济结构调整迈出大步伐。实施亿元以上调结构项目953个，项目集中开工成为常态。装备制造业、高新技术产业增加值增速分别高于规模以上工业6个和7个百分点，金融业增加值增长11%，规模以上服务业企业达到458家，第三产业占比提高2.1个百分点。大众创业、万众创新催生经济新活力，新增市场主体7.4万户、中小微企业1.3万户、科技型中小企业1020家，7家企业在“新三板”挂牌。农业发展方式加快转变，新培育新型农业经营主体1121个、增长17.2%。

三是着力抢抓京津冀协同发展机遇。把承接产业转移摆在突出位置，谋划实施亿元以上京津合作项目292项，累计完成投资800亿元。京津两大示范区合作共建扎实推进，京冀(曹妃甸)协同发展示范区签约北京项目75个，开工45个、总投资1376亿元，津冀(芦台·汉沽)协同发展示范区正式启动，协同发展成为推动唐山经济转型的重要动力。

四是着力推动沿海增长极“热”起来。借力京津加速临港产业聚集，以曹妃甸、海港开发区为龙头的沿海一线招商引资势头强劲，投资者关注度大为提升。曹妃甸石化产业基地、中日韩循环经济示范基地等一批产业园区快速发展，沿海板块谋划实施亿元以上项目707个、累计完成投资1089亿元。唐山港完成货物吞吐量4.9亿吨，集装箱152万标箱、增长37%。有史以来第一艘40万吨级货船驶入渤海湾，停靠在曹妃甸港区。张唐铁路全线通车，港口腹地得到拓展，唐山正在成为“一带一路”重要枢纽。

五是着力推进“三城联动”。统筹抓好城市建设、城市经济、城市管理，中心城区建成了一批标志性工程和基础设施，世园会会址建设全面提速，唐古快速路、老205国道全线贯通，城市功能和宜居水平进一步提升。加快发展总部经济、楼宇经济，涌现出天元、福地、高科等一批税收“千万元楼”。高新区等十大特色产业园区完成主营业务收入3144亿元，成为城市经济的第一支撑。

六是着力解决民生领域突出问题。积极回应群众诉求，切实办好顺民意、惠民生的实事好事，开展清理拖欠农民工和企业职工工资专项行动，下大力处置化解市中心区回迁房、商品房遗留问题及住宅小区“两证”规范办理等复杂矛盾。全面启动老交大片区搬迁改造，彻底消除震后简易房。面向社会开放学校体育场地，市中心体育公园、植物园、陶瓷公园建成开放，成为市民健身休闲娱乐的新去处。强力推进环保治理专项行动，全年空气质量达标天数同比增加23天，重度污染以上天数减少30天，环境空气质量改善率达到15.8%。

在推进经济社会发展的同时，更加重视和加强政府自身建设，认真践行“三严三实”，严格落实“八项规定”，深入开展解放思想大讨论和政府系统“爱唐山、做贡献”主题实践活动，形成了奋发作为、干事创业的强大正能量。设立政务工作微信群，搭建扁平化平台，政府工作效率和执行力明显提升。健全完善政府法律顾问制度，严格规范行政行为。自觉接受各方面监督，认真执行市人大及其常委会各项决议，办理人大代表建议244件、政协提案479件，按时办复率100%。

各位代表!过去的一年，我们在攻坚克难中打开局面，在团结奋进中凝聚力量，通过全市上下的不懈努力和扎实苦干，唐山大地发生了新的变化、焕发出新的活力，为“十三五”转型发展、跨越发展奠定了坚实基础。在此，我代表市政府，向全市人民，向人大代表、政协委员、各民主党派、工商联、无党派人士和人民团体，向驻唐部队、武警官兵、政法干警，向关心支持唐山发展的老领导、老同志，向所有为唐山发展做出贡献的同志们、朋友们，表示衷心的感谢并致以崇高的敬意!

在肯定成绩的同时，我们也清醒看到经济社会发展中存在的问题。一是经济下行压力依然较大，企稳回升基础还不稳固。二是转型升级任务艰巨，结构性矛盾突出，科技创新支撑作用不强，发展质量和效益有待提高。三是大气污染防治形势严峻，实现空气质量持续好转仍需付出艰苦努力。四是民生改善还有很多薄弱环节，社会建设管理、公共服务水平还需进一步提升。五是改革开放步伐需要全面加快，制约转型发展的体制机制障碍亟待破除。六是政府机关和工作人员思想作风有待改进，办事效率不高、服务意识不强，为政不廉、懒政怠政的现象不同程度存在。对此，我们一定用新的发展理念、改革创新的办法、务实管用的举措有效加以解决。

二、奋力开启唐山“十三五”转型发展新征程

“十三五”是唐山发展历史上重大机遇最为集中的时期，是各种优势潜力最能有效释放的时期，也是推动转型升级最为关键的时期。市委九届七次全会确立了我市“十三五”发展的指导思想、奋斗目标和基本要求。展望“十三五”，我们要牢记历史使命，朝着“三个努力建成”目标加速迈进。习近平总书记提出要努力把唐山建成东北亚地区经济合作窗口城市、环渤海地区新型工业化基地、首都经济圈重要支点。总书记提出这一目标五年了，我们做得怎么样，什么时候才能实现，这是唐山人必须回答的重大课题。未来几年，政府工作将全面聚焦“三个努力建成”，将各项任务分解落实到每个年度、每条战线、每个领域,努力把总书记为唐山描绘的蓝图尽早变成现实。我们要加快动力转换，坚决打赢转型升级这场硬仗。转型升级是唐山躲不开、绕不过的历史关口，是走出低谷、重振雄风的唯一出路。必须坚定革新图变的决心，在发展方式、动力转换上来一次大调整，加大创新驱动和供给侧结构性改革力度，尽快摆脱传统路径依赖，推动产业迈向中高端水平。只要跨过转型升级这道坎，唐山就一定能够迎来更加美好的前景。我们要抢抓难得机遇，把唐山的比较优势转化为发展优势。京津冀协同发展、“一带一路”、环渤海地区合作发展等机遇就在眼前，沿海开放、区位交通、产业基础等优势正在释放。抓实抓牢这些重大机遇，尽快唤醒沉睡的潜能，就能把别人羡慕我们的基础、条件和优势转化为加速崛起的动力，唐山就能在转型发展中闯出一片新天地。我们要直面困难挑战，在激烈竞争中突出重围、赢得主动。放眼沿海发达城市，唐山正面临标兵渐远、追兵渐近的双重压力;环顾省内其他地市，唐山正经受增速放缓、首位优势下降的严峻考验。在这种竞争格局中，我们只有增强责任意识、忧患意识、拼抢意识，付出比别人、比以往更多的努力，才能实现新跨越、再创新辉煌。我们要认真回应群众期待，以扎实的工作成效取信于民、造福于民。站在新起点，全市人民求富求强、求新求变，对政府工作满怀憧憬和希望，期盼我们把唐山发展得更好、建设得更美，共享做一名唐山人的幸福、尊严和荣光。我们只有殚思极虑、激情工作，把唐山发展提高到一个新的层次，才能让全市人民得到实惠、受到鼓舞、增强信心，我们的工作才能获得持久动力。

“十三五”时期全市经济社会发展的指导思想是：全面贯彻党的十八大和十八届三中、四中、五中全会精神，以邓小平理论、“三个代表”重要思想、科学发展观为指导，深入贯彻习近平总书记系列重要讲话精神，坚定不移践行创新、协调、绿色、开放、共享的发展理念，坚守发展、生态、民生三条底线，把握协同发展、转型升级、又好又快的工作主基调，按照市委九届七次全会部署，以“三个努力建成”为方向，扎实推进“32239”战略布局，坚持稳中求进，坚持以提高发展质量和效益为中心，着力深化供给侧结构性改革，统筹推进经济建设、政治建设、文化建设、社会建设和生态文明建设，奋力推动唐山转型升级、再创辉煌，确保如期全面建成小康社会，为努力建成东北亚地区经济合作窗口城市、环渤海地区新型工业化基地、首都经济圈重要支点和靓丽繁华宜居和谐的现代化沿海强市奠定坚实基础。

统筹考虑贯彻五大发展理念和唐山发展需要，“十三五”时期在保持地区生产总值增长的同时，把重点放在提升经济运行质量和效益上，努力实现“五个提高”：提高财政收入占地区生产总值比重、提高企业利润率、提高城乡居民人均收入水平、提高城镇化率、提高全要素生产率。主要指标共设定27项，其中约束性指标13.5项、预期性指标13.5项。总体目标为“三个领先、两个翻番、一个全面建成”，即：经济增长速度达到全省平均水平，转型发展成效全省乃至全国领先，地区生产总值年均增长6.5%，总量继续保持全省第一;发展迈入中高端，质量效益提升幅度全省领先，一般公共预算收入年均增长8%以上，到2020年达到492亿元;环境治理成效明显，空气质量改善程度全省领先，力争退出全国空气质量监测城市排名后十位。地区生产总值和城乡居民可支配收入比2010年翻一番，到2020年如期全面建成小康社会，“三个努力建成”主要指标基本完成，全力打造令人向往、世人尊敬的新唐山。

实现“十三五”发展目标，必须积极适应引领经济发展新常态，坚持穿新鞋、走新路，坚持变中求新、变中求进、变中突破，努力实现多方面工作重点转变，走出一条质量更高、效益更好、结构更优、优势充分释放的发展新路。

一是突出转型发展，努力走出一条以结构调整推动中高端发展的新路。唐山经济遇到的困难，虽有外部环境影响，但根子在于产业结构不合理，矛盾的主要方面在供给侧。围绕建成环渤海地区新型工业化基地，更加注重供给侧结构性改革，扎实做好“加减乘除”四篇文章，大力实施传统产业改造提升、新兴产业培育等十大工程和服务业赶超振兴计划，构建绿色低碳循环产业体系。到2020年，钢铁产业高附加值产品比重提高到30%以上，装备制造业增加值占规模以上工业的比重达到25%以上，战略性新兴产业增加值年均增长14%以上，服务业占地区生产总值的比重达到38%，农业产业化经营率达到70%。

二是突出创新发展，努力走出一条以“双创”引领产业变革的新路。创新是实现动能转换、引领唐山发展的第一动力。以争创国家创新型城市为契机，坚持技术与市场双轮驱动，在大众创业、万众创新上用实招、出亮点，引导创业者和市场主体专注于某一个领域，把主业做到极致、做出特色，以连环创业催生创新成果、释放创业活力，推动唐山产业“裂变升级”。强化企业主体地位，增加研发投入，推动先进装备制造、新材料等新兴产业做大做强。到2020年，全社会研发经费支出占GDP比重达到2.5%，科技型中小企业5000家以上。

三是突出协调发展，努力走出一条以补齐短板拓展发展空间的新路。解决发展不平衡、不协调、不可持续问题，是提升唐山整体竞争力的内在要求。围绕建成首都经济圈重要支点，全面融入京津冀协同发展格局，做大做强沿海、城市、县域三大板块。到“十三五”末，沿海板块经济总量和一般公共预算收入比2015年翻一番，中心城市建成区面积410平方公里以上、常住人口318万，更多县(市)进入全国百强。统筹城乡发展，全面提升新型城镇化水平，到2020年县城人口占全县人口40%以上，基本实现美丽乡村建设全覆盖。

四是突出绿色发展，努力走出一条以生态文明保障永续发展的新路。发展和生态如同一枚硬币的两面，两者相辅相成、缺一不可。牢固树立“绿水青山就是金山银山”理念，更加注重促进形成绿色生产方式和消费方式。科学设定并严格遵守资源消耗上限、环境质量底线和生态保护红线，制定实施自然资源资产负债表、生态离任审计、绿色发展负面清单等制度，坚决打好大气污染防治攻坚战和持久战，确保环境质量一年更比一年好。实施生态修复工程，到2020年森林覆盖率达到40%，国家森林城市、生态园林城市基本建成。治理大气污染，既是当务之急，也是一个长期艰苦的过程。只要我们坚定信心、精准发力、持续攻坚，就一定能够早日换回人民期盼的碧水蓝天!

五是突出开放发展，努力走出一条以改革开放激发发展活力的新路。沿海城市的本质是开放，唐山与沿海先进城市的差距也在开放。围绕建成东北亚地区经济合作窗口城市，坚持引进来与走出去并重，加快构建全方位、多层次的开放体系。到2020年，全市经济外向度达到30%;唐山港货物吞吐量达到6亿吨以上，集装箱力争达到500万标箱。坚持以开放促改革，下大力突破体制机制障碍，在重点领域和关键环节改革上取得决定性成果，力促经济社会发展活力得到充分释放。

六是突出共享发展，努力走出一条以改善民生提升幸福指数的新路。只有让群众共享发展成果，发展才更有温度，幸福才更有质感。优化财政支出结构，确保民生投入稳步增长。积极促进创业就业，完善社会保障体系，实施教育提质惠民工程，推进健康唐山建设，扎实办好民生实事，让改革发展成果更多更公平地惠及全市人民。到2020年，城镇、农村居民人均可支配收入分别达到43800元和19600元。

各位代表!展望“十三五”，我们站在更高历史起点上开启新的伟大征程，前景十分广阔，目标催人奋进。我们坚信，经过全市人民的共同努力，五年的宏伟目标一定能如期实现，唐山的明天一定会更加美好!

三、2016年工作安排

今年是决战“十三五”开局之年，也是唐山大考之年。我们将迎来纪念抗震40周年，举办世界园艺博览会、中国—中东欧国家地方领导人会议、中国—拉美企业家高峰会、中国·河北国际经贸洽谈会、金鸡百花电影节等系列重大活动，世界目光将聚焦唐山。做好今年工作，既关系“十三五”开局起步，也关系到能否向世人充分展示震后唐山建设的巨大成就和崭新变化。面对特殊年份和关键阶段，今年政府工作总体上把握“两个确保”：一是确保世园会等系列重大活动安全顺利精彩举办;二是确保主要经济指标平稳较快增长，在全省保持合理位次。

今年经济社会发展主要预期目标是：地区生产总值增长5%，一般公共预算收入增长6%，固定资产投资增长10%以上，社会消费品零售总额增长10%，出口总值增长6%，规模以上工业增加值增长4.5%。单位生产总值能耗下降率、化学需氧量、二氧化硫和氨氮、氮氧化物排放量均完成省达目标。城乡居民人均可支配收入增长7%。居民消费价格指数涨幅控制在3%左右。城镇新增就业9.4万人，登记失业率控制在4.5%以内。常住人口和户籍人口城镇化率分别提高1.9和2.2个百分点。需要指出的是，一般公共预算收入高于生产总值增幅，这是指标体系设定的一个重大转变，主要考虑提高发展质量效益，为调结构、转方式留出空间，实现调速不减势、量增质更优。

做好今年政府工作，一要更加注重发掘培育增量。没有增量，发展就没有后劲，转型就失去依托。围绕抓实“九大增长点”，大力实施项目攻坚、新兴产业培育、传统产业提升、成长性企业“登台阶”、旅游业倍增等37个专项推进任务，打好经济下行阻击战的组合拳，靠增量培育新的增长点。二要更加注重加强供给侧结构性改革。统筹把握去产能与稳增长、稳增长与调结构两个平衡点，全力推进技术、产品、业态创新，扩大有效供给，改善供给品质，不断提高全要素生产率，实现向高水平供需平衡跃升。三要更加注重激活企业发展内在动力。企业是转型升级的中坚力量。当前形势下，要格外关心企业的生存发展，为他们在经济下行的寒冬里注入“唐山暖流”，引导企业树雄心、创大业，在新常态下找到新出路、展现新作为。在政策扶持上，对外来和本地企业一视同仁、等量齐观，不能内外有别、厚此薄彼。四要更加注重统筹兼顾。今年各项工作任务繁重繁杂、交织交汇。我们既要推进经济持续健康发展，又要全力办好世园会等重大活动，还要关注和化解金融、债务、去产能、去库存等领域的风险和矛盾，必须用系统思维、统筹办法，确保把好事办好、大事办成、难事办妥，推动唐山发展行稳致远。五要更加注重谋实抓深、精准发力。认真落实“四个干”机制，精准量化目标，精准明责问效，精准督导考核，真正把各项工作谋到关键处、抓到点子上，让深入抓、具体抓、精准抓成为习惯和自觉。

按照市委“32239”总体思路，围绕上述目标要求，重点抓好以下12个方面工作。

(一)坚持以确保世园会等重大活动安全顺利精彩举办为统揽，全面提升唐山知名度、美誉度和影响力。举办世园会等系列重大活动意义非凡、影响深远，办得精彩唐山就能出彩。我们必须举全市之力、聚各方之智做好筹备举办工作，兑现向国际社会的庄严承诺。一是确保会址建设如期完成。2月底前世园会所有重点项目竣工，4月10日前展陈布展和景观提升全部完成。超前谋划“后世园会时期”场馆利用和运营管理，引入发展文化创意、会议会展、高端商务、舞台演艺等新兴业态，努力打造永不落幕的国际盛会。二是全面提升配套功能。强化三女河机场、铁路唐山站等通行保障能力，确保火车站西广场公交长途客运枢纽尽快竣工投用，抓好新华联酒店等一批商业、接待配套项目建设，健全完善长效保洁机制，以良好市容市貌迎接四海宾朋。三是精益求精做好服务保障。把安全作为最大底线，周密制定各类专项预案，抓好人员培训和实战演练，统筹重大活动衔接，搞好资源整合和综合利用，确保安全保卫万无一失、城市管理井然有序、服务接待全面到位、新闻宣传有声有色、社会氛围文明祥和。四是着力打造全民盛会。引导广大群众从我做起，争做志愿者、当好东道主，自觉为办好重大活动增光添彩。全面提升公交、餐饮、宾馆等窗口单位服务水平，加强出租车规范化管理，展示唐山市民和城市良好形象。世园会等重大活动是向世界展示唐山的平台和窗口，但归根结底要办成面向全市人民的惠民工程。坚持共建共享，通过场馆综合利用，调整、新设直通世园会园址的公交快线和旅游专线，使群众更便捷更充分享受世园会成果。

(二)坚持以稳增长为首要任务，突出抓好项目建设和增量培育。在经济下行压力持续加大、各种风险挑战不断增多的形势下，稳增长依然是唐山今年经济工作的重中之重。准确把握新常态下稳增长的政策导向，着力优化投资结构，积极扩大有效投资，加快培育新的经济增长点。一是强力推进项目建设。今年市级重点实施亿元以上战略支撑项目230项，年度计划投资1282亿元。紧盯项目建设全过程，续建保竣工、新开抢进度、前期促开工、签约抓落地，着力破解重大项目引进、落地、建设中的难题，确保唐钢高强度汽车板二期等110个项目早日开工建设，迁西景田百岁山北方生产基地等40个项目尽快投产达效，102国道城区段改线等874个基础设施项目早日发挥效益。加大项目考核权重和结果运用力度，充分发挥考核指挥棒作用。谋划实施一批成长性好、建设周期短、吸纳就业多的中小项目，新培育中小微企业1.32万家。二是着力提速开发区发展。全面聚焦项目招商，确保每个开发区发展实现新突破，成为名副其实的增长点。年内，开发区引进省外资金和直接利用外资分别达到500亿元以上和9亿美元以上;全面落实省级以上开发区(园区)考核评价办法，确保主营业务收入、税收等主要经济指标增速明显高于全市、全省增幅。三是全力扩大开放招商。围绕推动京津冀协同发展，以承接京津产业项目为主攻方向，打好“沿海”“京津”两张牌，吸引更多优质项目落户唐山，确保北京新能源环卫汽车等34个产业项目开工建设。全年实际利用外资增长5%、引进域外资金增长10%以上。大力实施“凤还巢”工程，吸引更多企业回乡创业发展，确保年内回归规模性企业100家以上，在县(市、区)税收中有明显占比。用足用好中韩、中澳自贸协定等优惠政策，积极拓展外贸市场，确保出口总值达到90亿美元。

(三)坚持以供给侧结构性改革为契机，加快推进工业提质增效升级。着眼于提高供给体系质量和效益，抓住国家出台去产能扶持政策为唐山钢铁等产业带来的重大机遇，有针对性地做好“加减乘除”四篇文章，推动我市产业结构“减负”前行、轻装上阵。年内，工业投资、技改投资均增长12%，新增规模以上工业企业80家以上。一是以培育壮大新兴产业增创发展新优势。抓新兴产业就是实实在在的调结构，也是释放供给端活力的重要抓手。过去钢铁、煤炭等传统产业形势好的时候，由于新兴产业占比小、不添秤，没有给予足够重视，致使新兴产业未能真正“兴”起来，这也是我市转型任务繁重艰巨的原因之一。现在对新兴产业的培育，已经到了非抓不可的时候。既要抓紧抓实，又不能急于求成。围绕装备制造、新能源、新材料等重点领域，有针对性地制定出台扶持政策，力促新兴产业加速崛起，尽早挑起唐山发展大梁。年内重点抓好石墨烯产业基地等100个新兴产业项目，高新技术产业增加值增长14%。把培育壮大装备制造业作为重中之重，确保增加值达到650亿元、占规模以上工业比重22%以上。二是以扶优劣汰为成长性企业打造发展新空间。把处置“僵尸企业”作为化解过剩产能的牛鼻子，紧盯国家去产能新政特别是职工安置、债务化解等扶持政策，通过兼并重组、债务重组乃至破产清算，实现市场出清。年内压减炼铁产能578万吨、炼钢产能450万吨。加快推进国丰钢铁搬迁等一批重点钢铁企业改造升级项目，鼓励支持成长性好的钢铁企业走高端化发展道路。开展实体经济企业降本增效专项行动，不断增强企业的盈利能力和发展后劲。成长性企业市场有空间、发展有潜力，当前形势下扶一把、促一下，就会驶入“快车道”、登上“新台阶”。年内重点扶持100家成长性企业发展壮大，打造主营业务收入超300亿元企业2家、超100亿元5家、超10亿元18家。三是以催生新技术新产品提升企业竞争力。新常态下，传统行业正经历破茧重生的前夜，产业变革孕育新的突破。从供给端入手，重点抓好100项技术改造、100项钢铁深加工、100项两化融合和100项新产品开发等“四个一百”项目，延伸产业链、提升价值链、优化供应链。四是以提升企业家素质激发内生动力。企业家是企业发展最核心的因素。坚持因人而异、分类施教，通过走出去、请进来等方式，强化素质拓展培训，增强培训的针对性、实效性，引导帮助企业家打开解放思想“总阀门”，主动对标先进，实现创新发展。三年内培训各类企业董事长、总经理及成长性企业高管人员3000人以上，今年培训800人以上。

(四)坚持以产业聚集为核心，强力推动沿海经济加速崛起。历经多年开发建设，我市沿海地区已进入大规模产业聚集的关键时期，必须推进工作重心转移，下大力抓一批有利于发挥港口优势、壮大临港产业的大项目、好项目、新项目，为唐山转型升级、跨越发展提供强力支撑。一是协同发展示范区要在产业项目上快见成效。曹妃甸协同发展示范区，突出抓好保利北斗、机械电子产业园等一批重点产业项目，全年投产超亿元项目20个以上、开工50个以上，确保纪念抗震40周年之前取得阶段性成果。津冀(芦台·汉沽)协同发展示范区，全面启动对接合作，先行试验区建设取得实质性进展。申请天津自贸区政策向曹妃甸片区延伸尽快落实到位。二是重大开放平台要提速突破。抓实国家级石化产业基地建设，力促曹妃甸千万吨级炼油项目尽快落地，确保浅海炼化一体化等8个项目全面开工，甲醇制高清洁燃料等一批项目投产达效。推动中日韩循环经济示范基地上规模、见成效，年内核心区新开工超亿元项目10个以上、签约日韩项目20个以上，博凯盛触显一体化等4个项目竣工投产。发展曹妃甸综合保税区进口商品连锁超市，新增注册企业100家以上，整车进口口岸获批，国际仓储物流中心等14个项目投入运营，全年进出口额同比增长67%。京唐港保税物流中心通过国家验收。三是港口贸易要提质增效。加快曹妃甸港区中物通用码头、西区一号二号港池和丰南港区起步工程建设，着力完善化工、LNG、集装箱等泊位功能，实现过境资源能源就地交易加工、转化增值。积极吸引优质船代、货代、船运公司聚集，推进集装箱多式联运。年内，唐山港货物吞吐量达到5.05亿吨，集装箱突破165万标箱、增长8%。加强集疏运体系建设，唐曹铁路建成通车，水曹铁路全面开工，遵曹公路加快实施。以张唐铁路开通为契机，启动包头、二连浩特等内陆港建设，拓展连接西北地区以及蒙古国通道。四是港产城教一体发展要尽快突破。优化唐山湾生态城空间布局，提速生态城大学城建设，华北理工大学暑假完成搬迁、9月份开学，保信集团职教城、翔宇职业技术学院等项目开工建设。以与北京共建现代化新城、争创“创客新城”为契机，加快3.5平方公里产城融合起步区建设，吸引北京医疗、科技、文化等公共服务资源聚集，景山学校曹妃甸分校按期开学，北京安贞、友谊、妇产医院合作办医开门接诊，为曹妃甸发展聚人气、增活力。

(五)坚持以拓展业态、做大规模为重点，全面促进现代服务业大发展、快发展。坚持双轮驱动、多点发力，推动生产性服务业向专业化和价值链高端延伸、生活性服务业向精细化高品质提升。年内新增规模以上服务业企业130家以上，服务业增加值增长7%。一是依托综合交通优势做强现代物流业。重点抓好唐山传化公路港等20个物流项目建设，培育壮大佳源集团、海港远大等与我市现代产业体系相配套的第三方、第四方物流企业，做大做强10个省级物流产业聚集区，加快打造国家级物流枢纽城市。全年新增规模以上物流业企业50家以上，物流业增加值增长8%以上、总量突破850亿元。二是围绕服务实体经济发展金融业。年内金融业增加值确保增长15%以上。加快发展融资租赁、股权投资、互联网金融等新兴业态，力促国家开发银行、平安银行在唐山设立分支机构。扶持壮大唐山金融中心，年内入驻运营率达到85%以上。支持唐山银行、唐山农商行、燕赵财险、金发集团等地方法人金融机构做大做强，力争总资产突破2900亿元。启动迁安、滦南农村信用社改制组建农商行。推动7家村镇银行延伸服务网点，实现县域全覆盖。三是利用“互联网+”提升传统商贸业。加速电子商务与我市产业深度融合，扩大工业产品、农产品、陶瓷等特色工艺品销售，确保交易在唐山、税收留在唐山。支持大型商场、连锁超市、便利店等零售企业发展O2O、定制化营销等模式，打造一批电商龙头企业，建成20个特色电商交易平台。推进农村电子商务全覆盖。加快发展邮政速递业。全面提升商务会展业发展水平，积极筹办京津冀户外用品博览会等特色展会，力争2016年中国食品餐饮博览会落户唐山。四是立足优质资源做大旅游业。借势世园会等重大活动的带动效应，引爆全市旅游业快速发展。年内旅游业接待人数、总收入分别增长30%和40%。整合链接开滦国家矿山公园、启新1889及陶瓷文化创意产业园等旅游资源，抓好清东陵、滦州古城等8大景区建设，打造10条精品旅游线路，推动南湖世园会园址创建5A级景区、国际旅游岛创建国家级旅游度假区。开通市区至重点4A级景区直通车，完成100座旅游厕所改扩建任务。培育10个省级乡村旅游示范点、30家星级乡村酒店和20种唐山特色旅游商品。五是着眼提升城市软实力激活文化健康产业。鼓励社会资本投资文化产业，通过设立“三枝花”剧场、主题酒店和文化创意主题街区、文化产业孵化器、网络文艺制作平台等形式，让唐山特色艺术焕发生机，催生新的业态。年内引进大型文化创意、影视制作、演艺娱乐等企业3家以上。加快发展赛事经济，拉动体育消费，引进赛事策划、体育产业运营企业3家以上。

(六)坚持以增强城市繁华度和宜居性为指向，不断提高“三城联动”发展水平。围绕打造京津冀区域中心城市和靓丽繁华宜居和谐的沿海强市，转变规划建设管理理念，推动城市发展由外延式扩张向内涵式提升转变，让广大市民充分享受自由舒朗的城市生活。一是推动城市经济激活提质增量。城市是经济活动的主要载体。坚持盘活引进、错位发展，抓实城市商圈和楼宇经济这个增长点，着力打造唐百大、八方购物、远洋城、硅谷、南湖生活休闲娱乐广场等5大商圈，培育50栋大型商务楼宇，年内纳税超2000万元的楼宇达到8栋以上、超5000万元的5栋以上、超亿元的2栋以上。大力推进钢结构住宅产业化。积极推动房地产去库存，进一步打通供需通道，稳定房地产市场。二是拓展优化城市公共空间。以完成城市规划修编为契机，突出惠民理念，谋划实施一批彰显城市魅力、丰富城市表情、留住唐山独特元素的重点项目，围绕老旧小区和居民密集区等场所，新建改造10个公园、游园、绿地及一批公共文化服务设施。谋划、建设和运营好唐山交大文化园，使之成为传承历史文脉、可赏可品可用的精品工程。依托世园会园址这个最大的城市公共空间，切实建好用好城市规划馆、低碳生活馆、少年世博园等场馆设施，把南湖打造成独具魅力的“城市会客厅”和旅游休闲目的地，让每一位市民都感到自己是城市的主人，拥有实实在在的获得感。三是完善提升城市承载能力。围绕让市民居住、出行、生活更加舒适便捷通畅，新建续建16条城区道路，二环路年内全线通车，市区新增集中供热能力636万平方米，完成53个老旧小区供水管网及市中心区47万平方米建筑节能改造，确保青少年宫、工人文化宫、科技馆等公共服务设施尽快投用。启动地下综合管廊项目。抓好海绵城市试点建设。依托唐车公司资源，谋划实施城市轨道交通。四是全面提高城市管理水平。大力实施绿美亮净畅工程，推进城市出入口、繁商区等重点区域景观提升，完成9条主干道街景整治、10条道路亮化改造，谋划实施过街天桥、地下人行通道、立体停车场等交通设施项目，加快建设“智慧唐山”，全面推广数字化城管模式，靠管理提升城市品位、彰显唐山特色，打造市民引以为豪、令人耳目一新、能够留住记忆的唐山新形象。

(七)坚持以破除体制机制障碍为着力点，扎实推进重大改革举措落地见效。全面聚焦转型升级，着力抓好中央和省各项改革政策的衔接落实，精准实施行政审批、国企、财税等重点领域改革，为经济社会发展增活力、添动力。一是深化行政审批制度改革。扎实推进“放管服”，坚决砍掉各类繁琐手续，向县(市、区)和省级以上开发区(园区)精简下放一批市级审批事项，全面推行多部门并联审批和协同办理，加快市级网上审批平台向县(市、区)、乡镇(街道)延伸，实现行政审批“瘦身”提速。整合建立统一的公共资源交易平台，不断提高交易效率和服务水平。推动行业协会商会与行政机关脱钩。二是深化国有企业改革。鼓励各类所有制企业参与国企改革，推进优势企业股权多元化改制，完善法人治理结构，盘活国有企业资产，实现增值增效。探索组建国有资本运营公司，稳步推进薪酬制度改革，促进国有企业焕发新的生机活力。三是深化投融资体制改革。大力推广PPP模式，推进政府融资平台市场化转型，化解政府存量债务。组建融资租赁公司，充分发挥股权投资、担保融资、财政贴息等撬动作用，助力基础设施和重点项目建设。制定工业园区道路及配套设施建设标准，形成有利于集约用地、节约投资的制度规范。四是深化社会事业改革。健全推广政府购买公共服务模式，积极推进生产经营性事业单位转企改制。深化城市公立医院综合改革，力争年内引入社会资本合资共建市中心医院实现开诊运营。6月底前完成市县公务用车制度改革。五是深化农村综合改革。农村土地承包经营权确权登记颁证累计完成全市承包地总面积的80%，市级农村产权流转交易中心实现运营，新建3至5个县级农村产权流转交易中心。组建市县两级供销合作集团，健全为民服务网络。

(八)坚持以大众创业、万众创新为引领，充分开掘经济发展新动能。进入转型升级的关键阶段，我们比以往任何时候都更加需要依靠创新创业聚集要素、培育动力。一是突出京津孵化、唐山产业化。盯住京津科技成果转化“最后一公里”，加大科技招商力度，建立科技创新产业化中试基地，打造京津科技成果转化首选地。年内，启动2至3个科创产业园建设，引进科技成果产业化项目(企业)50个。打造唐山钢铁创新谷，吸引大型知名企业入驻。二是突出企业创新主体作用。坚持市场驱动、集群发展，加大科技型中小企业培育力度，年内新增科技型中小企业500家以上。鼓励企业加大研发投入，提高自主创新能力，研发推广高速动车组结构轻量化等一批共性关键技术。全社会研发经费支出占GDP比重增长20%以上。三是突出科技创新服务。完善提升高新区创业中心等孵化器功能，新增市级以上科技企业孵化机构10家、中小企业技术创新公共服务示范机构3家、研发机构18家，省级以上院士工作站达到21家。四是突出培育“双创”工作新亮点。实行灵活动态的分段奖励政策，鼓励支持企业上市融资发展，全力打造资本市场“唐山板块”，年内新增“新三板”及以上上市挂牌企业30家。制定切实可行的政策措施，打造创业空间，积极发展众创、众包、众扶、众筹等新模式，激发全社会创新创业活力，新登记市场主体7万户以上，万人拥有企业户数达到100家。五是突出创新人才支撑。健全完善培养、引进、激励人才的政策措施和动态机制，吸引更多行业领军人才、科技拔尖人才、创新创业团队和优秀毕业生来唐创业发展。充分利用我市高校、职业技术学校等资源，着力培养一批企业欢迎、市场需要的实用型技术人才，壮大“蓝领军团”。

(九)坚持以提升质量效益为目标，着力推动现代农业发展上水平。用工业理念、市场思维，加快发展高效、优质、生态、品牌、安全农业，使农业真正成为既富民又强市的产业。年内，农业产业化经营率达到68%。一是大力培育农业产业化龙头企业。抓好滦州农业科技产业园等200个投资千万元以上重点项目建设，市级以上农业龙头企业达到390家以上。积极创建国家农产品质量安全市。实施农产品品牌战略，争创中国驰名商标2件、省著名商标6件、地理标志证明商标3件、地理标志保护产品2件。二是全面提升现代农业园区功能效益。突出资源优势和产业特色，打造一二三产融合、产加销游一体、产业链条完整的现代农业园区。年内，争创省级园区5个、新认定市级园区20个，新发展专业大户140个、示范家庭农场40个。三是加快实施山区综合开发。山地丘陵占全市总面积的三分之一，山区综合开发对唐山意义重大，是打开发展空间的重要切入点。引入工商资本投资山区开发，培育多类型开发经营主体，实施果品基地、林下经济、观光旅游三大工程，建设6个市级山区综合开发片区，打造10个省级示范园区和经济沟，高标准山区综合开发面积达到6万亩以上。四是健全完善农业服务体系。创新金融、科技等惠农支农方式，推进农业股权投资，扩大“政银保”合作贷款，年内培育新型职业农民2000人。开展“互联网+”现代农业行动，加强与阿里巴巴、京东等电商平台合作，开办网上唐山特色农产品馆。携手北京“农管家”等农技服务供应商，建成覆盖10万户以上的农技服务平台。五是持续推进美丽乡村建设。开展道路硬化、改水改厕等12个专项行动，完成403个省级美丽乡村重点村建设，突出打造10个重点片区，建成10个中心村、20个精品村、30个旅游村。

(十)坚持以县域经济为支撑，全力加快新型城镇化和城乡统筹发展。深入推进以人为核心的新型城镇化，实现扩大规模和提高质量相统一，人口聚集与产业壮大相协调，全面提升县域板块发展水平。一是推进县域经济增比进位突破。坚持产城教融合发展，工业园区、农业园区一体推进，每个县(市)集中发展1至2个特色产业集群，培育一批龙头企业和区域品牌，推动县域经济快速增长。打造一批旅游服务、商贸物流等特色镇，提升县域整体实力。二是推进县城扩容升级提质。扎实开展县城建设三年攻坚行动，加快实施一批公共服务和基础设施项目，年底全市县城建成区总面积达到183平方公里，所有县城实现集中供暖、集中供水和污水处理，全部建成数字化城管。三是推进县际交通基础设施互联互通。优先打通缺失路段，畅通瓶颈路段，彻底解决县际之间不连不通、连而不通、通而不畅等问题。重点抓好总投资153亿元的迁曹高速、唐廊高速唐山段等高速公路建设，加快赤曹线等项目建设进度，谋划推进唐秦高速、京曹高速等项目，增强发展支撑能力。四是推进农业转移人口市民化。抓住提高户籍人口城镇化率这个关键，推进社保、医疗、就学等公共服务均等化，促进有能力在城镇稳定就业和生活的农业转移人口举家进城落户。年内转移农村人口18万人，户籍人口城镇化率提高到36.2%。

(十一)坚持以环保治理攻坚为抓手，下大力改善生态环境质量。抓好大气污染防治，事关群众切身利益，事关唐山对外形象，事关世园会等重大活动成效。必须向环境污染持续宣战，用硬措施完成硬任务，确保空气质量持续改善。一是下大力破解污染围城局面。加快城区及周边重污染企业搬迁改造，按时限完成唐钢焦化5#和6#焦炉、征楠焦化、新兴焦化、国丰钢铁北区关停搬迁。实施挥发性有机物治理，大力推动焦化、水泥行业脱硫脱硝和除尘设施全面升级，完成钢铁、电力等四大行业露天堆场封闭改造。取缔城市外环线内所有经营性散料堆场，外环线外散料堆场进棚进仓。二是下大力治理燃煤污染。坚持控煤与替代并重，严控新增燃煤项目，市中心区域彻底结束燃煤历史，其他区域全面取缔10蒸吨以下燃煤锅炉。年内削减煤炭消费量200万吨，低品位工业余热供暖面积达到1000万平方米，努力把燃煤污染降到最低。三是下大力抓好点面源污染治理。加强网格化、精细化管理，狠抓道路工地扬尘、机动车尾气、焚烧秸秆垃圾、露天烧烤、餐饮业油烟排放等污染治理。加快老旧汽车更新，率先实施市中心区公交、环卫等新能源汽车替代。四是下大力实施生态修复。开展“清水、净土、增绿”行动，狠抓水、土壤污染防治及造林绿化。强化非金属矿山治理，严控新增采石场，对281家露天采石场实施覆土复绿整治，让矿山披上生态绿衣。创建国家森林城市，推进绿色唐山攻坚，新建绿化示范村400个，全市新增造林面积35.7万亩，森林覆盖率达到36.6%。

(十二)坚持以保障和改善民生为根本，让人民群众有更多获得感。民之所望，施政所向。尽管经济下行和财政收支压力很大，但改善民生力度不能减弱、投入不能减少。今年，市财政安排民生和社会事业资金94.9亿元，占市本级可用财力的75.3%。拓宽创业就业渠道。落实援企稳岗政策，统筹解决好高校毕业生、农村转移劳动力、化解过剩产能失业人员等重点人群就业问题。织牢社会保障网络。实施“全民参保登记计划”，推动实现从制度全覆盖向人员全覆盖。城市低保标准由每人每月500元提高到550元，特困人员供养标准由每人每月500元提高到825元，建设保障性安居工程1.8万套，加强贫困残疾人福利保障。促进教育公平发展和质量提升。在全省率先实现所有县(市、区)义务教育均衡发展达到国标。改造农村义务教育薄弱校100所，高中阶段教育毛入学率达到93.7%。重视和加强学前教育，强化公办幼儿园建设和幼儿教师培养，新改扩建幼儿园60所，唐山幼儿高等师范专科学校挂牌招生。推动高等教育提质增效。提升职业教育服务转型发展能力。积极推进“百所学校对接京津行动”，持续开展名师、名校、名校长工程，全面提高教育竞争力。实施文化体育惠民工程。加强基层公共文化设施建设和管理，精心组织世园会文化展演、评剧艺术节、金鸡百花电影节等文化活动，继续办好“文化大讲堂”和电影惠民放映活动，以健康文明引领社会新风尚。唐山大剧院上半年投入使用、发挥效益。全面完成市体育中心场馆改造提升，建设一批小型公共运动场。广泛开展全民健身运动，积极筹办中国乒超联赛(唐山)、南湖马拉松、自行车赛等赛事活动，打造高水平、国际性特色体育赛事品牌。推进健康唐山建设。健全基层卫生服务体系，深入实施“分级诊疗”制度。建立市级药品集中招标采购平台，减轻群众用药负担。所有三级医院与京津名院开展实质性合作，二级及以下医院与京津医院实现远程会诊。抓好全面放开二胎政策落实。积极回应群众关切。继续办好惠民实事。认真做好信访工作，畅通利益诉求渠道，让百姓诉求有人听、有回应、有结果、有反馈。加快市中心区居民小区“两证”规范办理，妥善解决回迁房、商品房遗留问题，让回迁群众尽早结束在外漂泊、靠租房度日的生活。维护社会和谐稳定。深化“平安唐山”建设，严厉打击违法犯罪活动。开展重点领域安全生产整治，坚决遏制重特大事故发生。创建国家食品安全城市，提高食品安全保障能力和水平。加强全民国防教育和后备力量建设，做好双拥优抚、人口计生、民族宗教、外事侨务、广播电视、妇女儿童、老区建设、防震减灾、气象、档案等各项工作。

四、树立新理念，激发新状态，进一步凝聚推动唐山转型升级、再创辉煌的强大合力

迈上新起点，开启新征程，政府工作必须有新作风、新状态。我们一定按照忠诚、干净、担当、实干的要求，恪守为民之责，践行“三严三实”，勇担发展重任，用新理念引领新发展，用新状态干出新成效，奋力开创各项工作新局面。

一是立足岗位，尽心尽责，让爱唐山、做贡献成为政府工作人员的价值追求。始终把职责记在心上、扛在肩上、抓在手上，人在岗上，岗在心上，把全部精力投入到谋事干事干成事上来。时刻反思这一天、这一周、这一月、这半年、这一年，自己为唐山干成了哪些事，是增了光、添了彩，还是延误了唐山发展，真正为干成事而自豪，为碌碌无为而不安，为影响发展而羞愧。

二是提振精神，奋发作为，让夙兴夜寐、激情工作成为政府系统运转常态。从市政府做起，沉下心，不浮躁，不懈怠，引导和动员各条战线、各个层面动起来、紧张起来，以只争朝夕的精神干工作、抓发展、促落实，坚决反对四平八稳、拖拖沓沓。特别是对重点项目、重大事项、重要工作，快谋划、快上手、快实施，把“一线”变成“前线”，把“前线”变成“火线”，用全体政府工作人员的辛苦指数换取全市人民的幸福指数。

三是转变作风，狠抓落实，让实干兴唐、创先争优成为政府工作的主旋律。按照“四个干”机制，对每项工作细化量化具体化，跟踪督导、定期通报、到期交账，确保事事有回音、件件有结果。坚决克服工作一般化、低标准倾向，高起点站位，高标准落实，推动各项工作实现新突破。坚持以开阔胸襟、真诚态度服务市场主体，大力整治慵懒散拖、推诿扯皮及“办证多、办事难”问题，让企业和群众少跑路就能办好事、不求人就能办成事，打造安心、舒心、放心的发展环境。

四是依法行政，从严治政，让遵纪守法、清廉自律成为政府工作人员的基本准则。自觉接受人大依法监督、政协民主监督和社会舆论监督，认真办理人大代表议案、建议和政协委员提案，推进政府重大决策科学化、民主化、合法性审查机制。带头学法尊法守法用法，始终做到心有所畏，言有所戒，行有所止。严格控制机构编制，确保财政供养人员只减不增。大力推行政务公开、行政权力公开，强化重点领域、关键环节的行政监察和审计监督。始终把纪律和规矩挺在前面，严格落实廉政建设责任制，带头遵守“八项规定”，坚决反对“四风”， 严肃查处违法违纪案件，坚决纠正损害群众利益的不正之风，着力营造干部清正、政府清廉、政治清明的良好风尚。

各位代表!激情成就梦想，实干创造未来。唐山是一座英雄的城市，勤劳智慧的唐山人民曾经创造了一个又一个奇迹。站在新起点、踏上新征程，一定能够在转型升级道路上不断创造新辉煌。让我们紧密团结在以习近平同志为总书记的党中央周围，在省委、省政府和市委的坚强领导下，在市人大、市政协的监督支持下，紧紧依靠全市人民，锐意进取，奋发作为，为全面建成小康社会、实现“三个努力建成”目标，加快建设靓丽、繁华、宜居、和谐的沿海强市而努力奋斗!
